# Supplementary material for: Reconstructing contact network structure and cross-immunity patterns from multiple infection histories
Source: PLoS Comput Biol. 2021 Sep 15;17(9):e1009375. doi: 10.1371/journal.pcbi.1009375 (PMC8475980; doi:10.1371/journal.pcbi.1009375)
Supplement: S1 Text — Fig A. Final epidemic size (in percent) for single genotype infections on dispersed (red) and clustered (blue) networks. Fig B. Genotype diversity index, multiplicity of infection (MOI), and epidemic prevalence for random regular graphs for five cross-immunity patterns. Fig c. Spatial correlations for clustered networks for five cross-immunity patterns. Fig D. Spatial correlations for dispersed networks for five cross-immunity patterns. Fig E. Correlation tests between a matrix based on infection barcodes distance and the network’s adjacency (top) or shortest path (bottom) matrices. Fig F. Mining for motifs of length two. Fig G. Spatial auto-correlations for dispersed (left) and clustered (right) neworks. (PDF) [file pcbi.1009375.s001.pdf]

Supporting information for: Reconstructing contact network structure and cross-immunity patterns from multiple infection histories

Christian Selinger[1,*], Samuel Alizon[1]

1 MIVEGEC, Univ. Montpellier, CNRS, IRD, Montpellier, France.

* christian.selinger@ird.fr

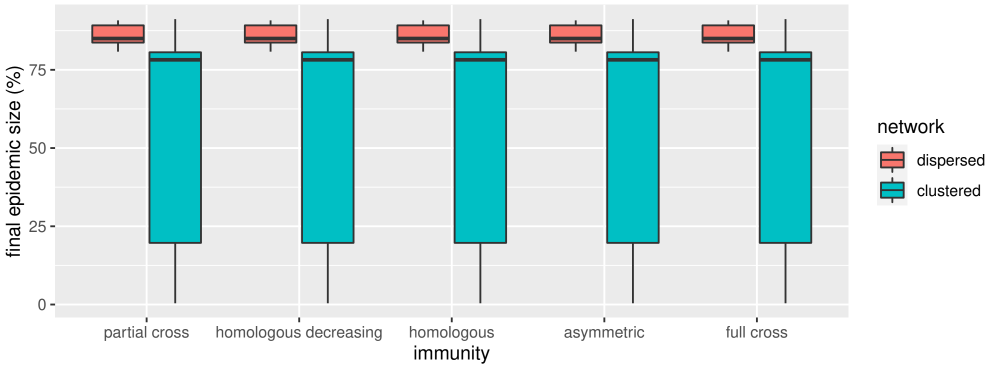

Fig A Final epidemic size (in percent) for single genotype infections on dispersed (red) and clustered (blue) networks. As expected for single infections, immunity assumptions do not impact the results. Median final epidemic size for dispersed networks are larger (85%) than for clustered networks (78%), while interquartile ranges are larger for clustered networks.

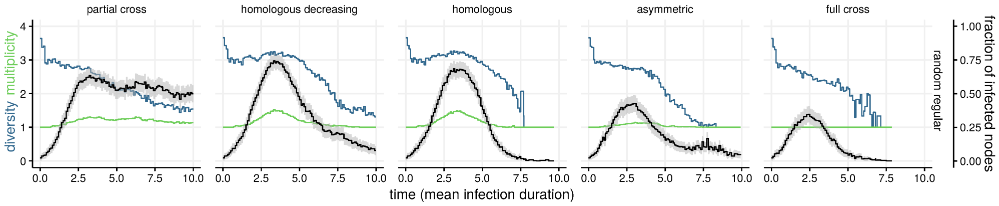

Fig B Genotype diversity index, multiplicity of infection (MOI), and epidemic prevalence for random regular graphs for five cross-immunity patterns. The figure shows the output of 50 stochastic epidemics with four genotypes on random regular graphs (with constant degree of 4, degree assortativity of 0, degree dispersion of 0 and average clustering of 0.0104), and five immunological interference settings. Black lines show the time-averaged fraction of nodes infected with at least one genotype (95% confidence intervals shaded grey), blue lines show the average genotype diversity index at the population level, and green lines show the average MOI of individual nodes.

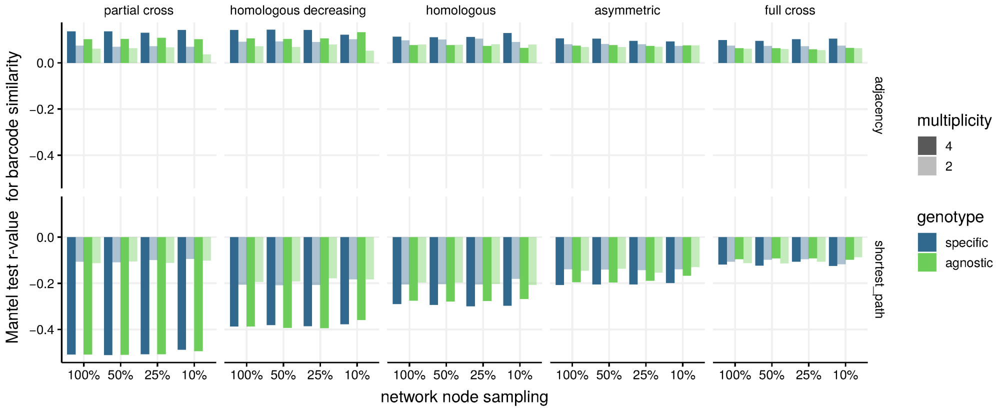

Fig C Spatial correlations for clustered networks for five cross-immunity patterns. Spatial correlations between an infection barcode similarity and the network's shortest path and adjacency matrix. We simulated epidemics with 2 or 4 circulating genotypes and for each combination of immunity setting for clustered networks, we re-sampled 20 times randomly $100, 50, 25$ or 10% of infected nodes and report the average r-value.

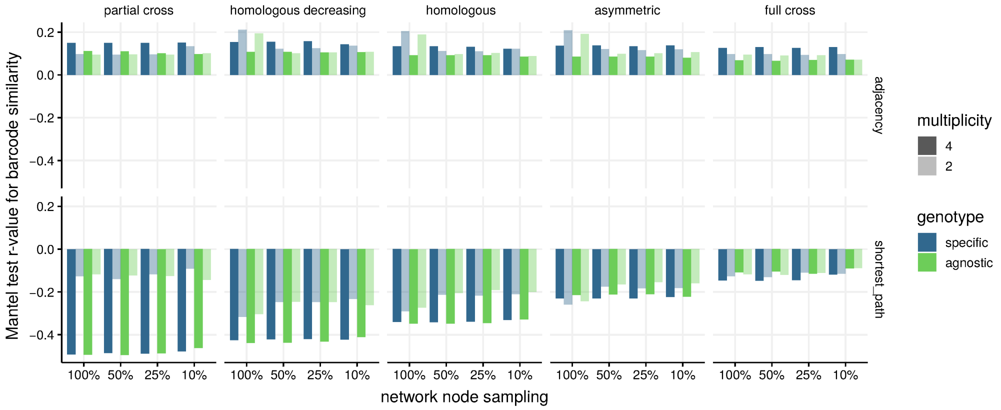

Fig D Spatial correlations for dispersed networks for five cross-immunity patterns. Spatial correlations between an infection barcode similarity and the network's shortest path (or adjacency) matrix on dispersed networks. We simulated epidemics with 2 or 4 circulating genotypes and for each combination of immunity setting, we resampled 20 times randomly 100, 50, 25 or 10% of infected nodes and report the average r-value.

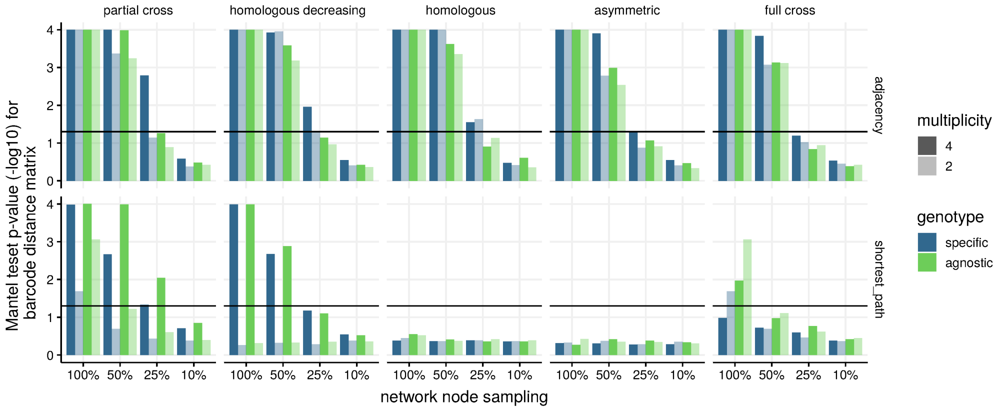

Fig E Correlation tests between a matrix based on infection barcodes distance and the network's adjacency (top) or shortest path (bottom) matrices. For simulated epidemics on clustered networks with 2 or 4 circulating genotypes and a variety of cross-immunity settings, we tested for correlations using p-values of two-sided Mantel tests with 10^4 permutations. For each setting, we re-sampled 20 times randomly 100, 50, 25, or 10% of the infected nodes and report the average p-value.

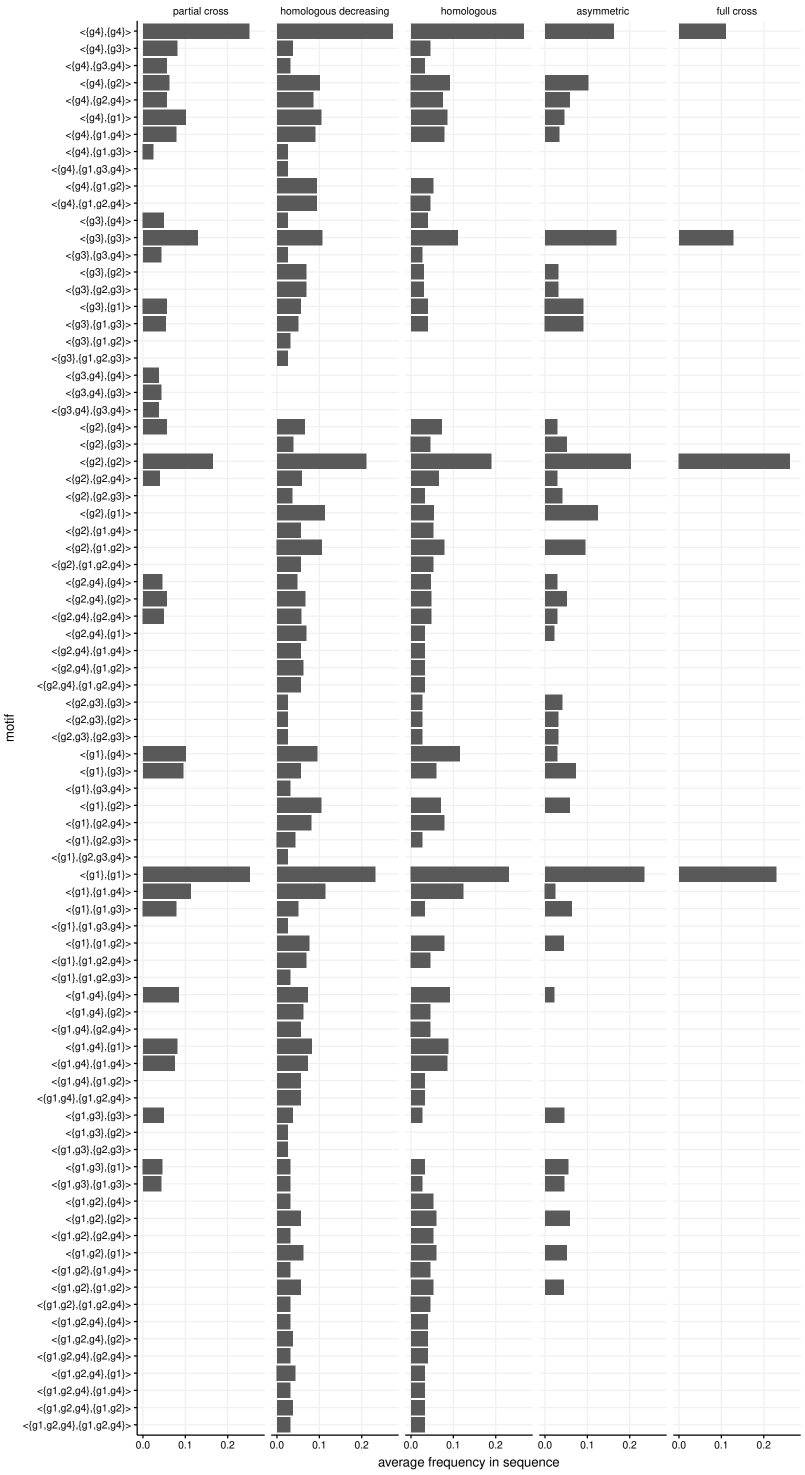

Fig F Mining for motifs of length two. Frequency of motifs with length two are displayed for five immunity settings, averaged across stochastic replicates.

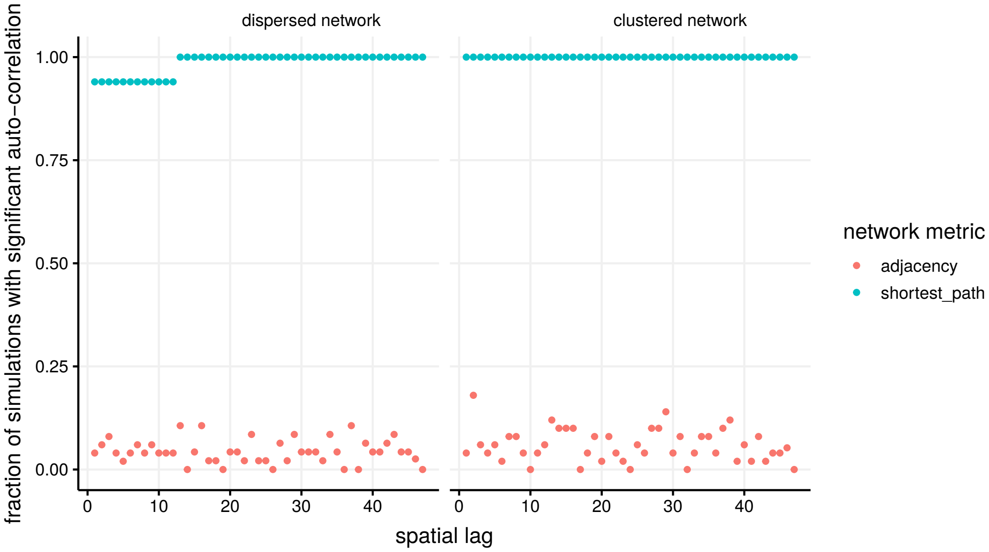

Fig G Spatial auto-correlations for dispersed (left) and clustered (right) neworks. The y-axis shows the fraction of simulations with significant spatial auto-correlations in adjacency (red) and shortest path (blue) matrices.
