# Supplementary material for: Reconstructing contact network structure and cross-immunity patterns from multiple infection histories
Source: PLoS Comput Biol. 2021 Sep 15;17(9):e1009375. doi: 10.1371/journal.pcbi.1009375 (PMC8475980; doi:10.1371/journal.pcbi.1009375)
Supplement: Attachment — Submitted filename: response_to_reviewers2.pdf [file pcbi.1009375.s002.pdf]

Responses to Reviewer #1's concerns

In this study the authors show that multiple infection histories of individuals can be used to identify characteristics of the underlying transmission network structure and that they can also be used to detect immunological interference between pathogens. They do this by developing a simulator of a stochastic epidemic model consisting of multiple infections on a network and introduce the idea of 'infection barcodes' (taken from barcode theory in computational topology) that encodes individual infection histories in the form of times of infection onset and recovery, and the type of pathogen.
They are able to show that the similarity matrices obtained by comparing infection barcodes between pairs of individuals are correlated with network adjacency matrices and that an individual's degree of connectivity in the network can be inferred from their connectivity in the space of the infection barcodes. Furthermore, they also show that analyzing pathogen co-occurrence patterns within hosts can be used to detect immunological interference.
Overall, I find the proof of concept described in this work to be very interesting and useful as the transmission network plays an important role in the spread of epidemics but it is generally very difficult to characterize. The methodology used in the paper is clear and I only have a few minor comments.

Thank you for this accurate and positive assessment of our work!

- Fig 1: x-axis is missing units of time
  We added a time unit in mean infection duration.
- Fig 2: I think the results of this section would become clearer if the Mantel test r-values were also added to this figure. Also, on first glance I found it a little confusing to see the correlation being positive for the adjacency matrix (in Fig.S1) but negative for the shortest path. Might help if this difference is described briefly in the Methods.
  We thank the reviewer for this comment. We added the following clarifications to the Methods section to give an intuition for the directionality of the r-values:
  $\rightarrow$ *"For the shortest path distance, we expected negative correlations with barcode similarity, since the further two nodes are apart from each other (i.e. the longer the shortest path distance), the less infection barcodes would be similar to each other. The converse holds for the adjacency, since adjacent nodes with unit weight would be similar in terms of infection histories."*
- Line 18: 'not only' might be missing between 'that' and 'the average'
  We thank the reviewer for this precision and corrected the sentence.
- Line 27: Opening quotation mark on 'contact'
  We made the change.
- Line 65: Opening quotation mark on 'age'
  We made the change.

- Line 104: 'Upon network initialization, we randomly seeded outbreaks of one infection per genotype, allowing for up to four genotypes per host.' Given that the outbreaks were seeded at the same time, how do you expect the results to be affected when the seeding occurs at different points in time? Naively I would expect this to decrease the difference between the genotype specific and the genotype agnostic case.
The reviewer is correct in that we performed simulations with simultaneous seeding of different genoytpes. Depending on the immunity setting, this should lower diversity and multiplicity dynamics in the beginning of the epidemic, since a founder strain can dominate subsequently seeded strains. Since richer (more diverse) multiple infection histories make correlations between barcode metrics and network metrics stronger, we expect sequential seeding to provide weaker correlations, unless we limit our sampling to time points where most the strains are circulating. This is now clarified in the text with the following sentence:
$\rightarrow$ *Upon network initialisation, we randomly seeded outbreaks of one infection per genotype simultaneously, allowing for up to four genotypes per host at high multiplicity.*

- Line 138: 'To better handle the computational workload of multiple infections on networks, we stored the infection histories in an interval tree data structure containing the times of onset and end of each infection episode, the genotype, and the host number'. In reality there might be uncertainties in the times of onset and end of infection episodes or the knowledge of infection episodes for individuals might be incomplete. How sensitive are the results to such uncertainties?
We thank the reviewer for this important remark. The potential impact depends what assumptions on the uncertainties are made. Our results derived using Mantel tests are certainly robust towards Gaussian errors, given zero mean and the permutation nature of the test. If episodes are incomplete, then multiple imputations approaches could be used. We added the following statement to the discussion section:*Also, real-world multiple infection histories might be incomplete or noisy. Determining how sensitive statistical associations between barcode similarity and network properties are, could help delineate practical limits of our approach.*

- Lines 149-157: Line 149 for example says, 'In the case of recovery of host i from infection with genotype g, we wrote the end time of the infection episode to the interval tree, removed the recovered infection from the rate vector, and added potential infections with genotypes other than g from the host's network neighborhood.' This implies that an individual can't get reinfected by g once they have recovered. Was this written just as an example? As for instance this is not the case for all the genotypes in immunity setting I_2.
We thank the reviewer for this clarifying question since from the initial formulation it indeed seemed that reinfection was impossible, which is not the case. We changed the sentence to:

→ *"In the case of recovery of host i from infection with genotype g, we wrote the end time of the infection episode to the interval tree, removed the recovered infection from the rate vector, and added potential infections with genotypes including g from the host's network neighbourhood."*

- Line 161: Opening quotation mark on 'clustered'
  We made the change.
- Line 320: 'Cross-immunity assumptions allowing for multiple infections with highly diverse infection barcodes (e.g. full cross-immunity, asymmetric decreasing) resulted in higher significance levels'. Is there a typo in this line? As it seems that the first three columns in Fig.2 (i.e. apart from 'full cross-immunity' and 'asymmetric decreasing') have higher significance levels.
  We thank the reviewer for pointing out this typo. We now mention the first three scenarios, which have higher diversity index and are more significant:
  → *"Cross-immunity assumptions allowing for multiple infections with highly diverse infection barcodes (e.g. partial cross, homologous decreasing, homologous) resulted in higher significance levels."*
- Line 325: 'We found that the correlations were highly significant even at a network sampling rate of 10'. Looking at Fig.2 this sentence comes across a bit strong, specifying the cases for which this is true will help.
  We agree that we overstated our results for the general case and, therefore, added the following specifics to the sentence:
  → *"We found that the correlations were highly significant e.g. for partial cross and homologous immunity down to a network sampling rate of 10%,..."*
- Line 322: 'Significance levels and correlation values appeared to be higher when tested for global network properties given by the shortest path matrix (bottom), which is consistent with the barcode similarity being a quantitative measure.' Is this statement also only restricted to the results in the first three columns of Fig.2? Also, is there an intuitive reason why the significance levels are so low (even for 100% sampling) for the shortest path full-cross immunity case? **We thank the reviewer for this observation. In contrast to adjacency matrices, the shortest-path distances go beyond the graph neighbourhood, consequently we generally expected that correlations between barcode similarity and network metrics are less strong. In particular, for the cross immunity setting at low multiplicity we lack sufficiently rich infection histories to distinguish between say distances of 3 vs. 4 towards a focal host. We added the following paragraph:**
  → *Likewise, when tested for global network properties given by the shortest path matrix (bottom), significance levels and correlation values appeared to be strongest for the same set of immunity assumptions. Overall, correlations with shortest path matrices were consistently less robust towards multiplicity than those with adjacency matrices (especially for the full cross immunity setting). This suggests that in order to distinguish distances between hosts beyond their graph neighbourhood with barcode similarities, one needs sufficiently rich multiple infection histories.*

Response to Reviewer #2's concerns

Overview

Selinger & Alizon present a wonderfully well-written manuscript and simulation study to explore how multiple infection histories can be used to infer network properties and immunological interference among co-circulating pathogens. This paper was justified, with very clearly written methods, and intuitive results. I have a few "major" comments, although they are more questions out of curiosity than necessary items to implement.

Thank you for this positive summary!

Major comments

- Have the authors considered differences in susceptibility across hosts? For example, younger individual may be more susceptible to infection with influenza due to less (or no) pre-existing immunity. This could also be the case for individuals with co-morbidities or individuals that are immunocompromised. Could these types of individual-level differences be implemented in this model?
The reviewer is correct in that differences in susceptibility are a major cofactor when studying multiple infections (the whole field of HIV coinfections is an illustration of this). There are two ways this could be implemented in the model for future studies. The first would be to have host heterogeneity be independent from the network structure itself. The goal would then be to test whether the inferences made regarding the network structure are robust to known host properties affecting susceptibility to infections. A second research axis could be to consider scenarios where infection by one of the parasites (or strains) affects host susceptibility to another parasite/strain. This latter scenario could be extremely rich because one could then expect an interaction between the structure of the network and the spread of first parasite.
→ *We added a paragraph in the discussion to explain the issues raised by host heterogeneity and how they raise particularly interesting subsequent questions.*
- I am wondering if the authors considered including a conceptual diagram for the barcode concept. I think this could be very useful to include in the methods, especially for those readers that are not familiar with barcode theory (probably most readers and infectious disease dynamics folks).
This is indeed a good suggestion to maximise the audience of the article.
→ *We added a conceptual diagram showing explicitly the workflow and the main idea of infection barcodes within networks.*
- Throughout the results, it would be very helpful to contextualize the findings more. Specifically, I think the authors could explain if results were intuitive or not; they do this to some degree, but I think even more would be very useful. For example, did the authors expect the partial cross-immunity scenario to most accurately predict node degree? If so why or why not? **We have contextualized at several instances**

the results (e.g. Reviewer 1's comment on line 322 on the directionality of r-values, reviewer 2's comment on Figure 1). As for the question why for the partial cross immunity barcode connectivity best predicted node degree, we added the following statement: *For this particular immunity setting, the area under the curve was* 0.74*, indicating that predictive power increased when using multiple infection data, especially because in this case diversity was maintained over the entire time horizon yielding highly specific barcode similarity scores.*

Minor comments

- Line 27: Could you add one reference for each: definition issues and assessing the appropriateness of various types of data?
  We added references to Antonovics 2017 and Arthur 2017 for definition and various data sources regarding contact networks.
- Line 38: upon first definition, it is not immediately clear to me what "taking advantage of multiple infection as a unique descriptor of a host's position within the network means." Could you please clarify this?
  We restructured the paragraph to clarify what we meant and also to give a more intuitive idea of our hypothesis.
- Line 41: although I agree that the norm is for longitudinal data to be used in more simple statistical models rather than dynamical models, there are a few very nice recent works that incorporated longitudinal data into individual-level dynamical models (Ranjeva et al. 2017- PMID: 29208707 and Ranjeva et al. 2019- doi: 10.1038/s41467-019-09652-6).
  We thank the reviewer for pointing out those two interesting references. We added them to the manuscript.
- Line 61: upon first introduction, I don't know what barcode theory is. It would be helpful to provide a very brief definition of barcode theory upfront in the introduction. **We added some concrete details to barcode theory in the introduction:** *To compare these infection histories, we use barcode theory inspired by computational topology [Edelsbrunner et al. 2010], where sets of intervals -in our case infection onset and clearance- are compared between each other.*
- Fig 1. I am surprised that diversity decreases as much in the homologous setting as in the full cross-immunity setting? Is this expected? It might be nice to quantify the differences between panels in Figure 1... like are the diversity, MOI, and time-averaged fraction of nodes infected statistically significantly different across panels?
  Diversity appears to decrease initially in all the runs, which is likely due to stochasticity (some strains grow more than others). The striking differences in diversity appears later on with three groups with distinct shapes: (i) partial cross-immunity maintains moderate diversity throughout, (ii) homologous and homologous decreasing have diversity following the fraction of infection nodes, with a sharp increase and decrease towards the end of the simula-

tion, and (iii) asymmetric and full cross-immunity show continuously declining diversity. Since performing statistical comparisons between the scenarios based on summary statistics for the aforementioned shapes is complicated, we added more descriptive details: *Diversity decreased initially in all the runs, which was likely due to stochasticity (some strains grow more than others). The striking differences in diversity appeared later on with three groups with distinct shapes: (i) partial cross-immunity maintained moderate diversity throughout, (ii) homologous and homologous decreasing immunity had diversity following the fraction of infection nodes, with a sharp increase and decrease towards the end of the simulation, and (iii) asymmetric and full cross-immunity showed continuously declining diversity.*

- it would be helpful in Figure 5 to include the immunity input matrix too, maybe there could be one additional panel with the 5 immunity input matrices shown, this would allow an easier comparison between co-occurrence score and the potential (or lack thereof) for homo / heterologous protection
  The matrices are shown in page 4 and the colour code largely reflects their structure (with red values corresponding to 100% cross-immunity. However, we agree that the caption was not very helpful.
  $\rightarrow$ *We edited the caption to point to the underlying matrices and to indicate that the color code largely reflects the matrix structure.*

Response to the Editor's concerns

- The Author Summary is well-written but it might be a little too technical for the intended audience
  We removed some of the technical terms in the summary and replaced them by more explicit wording.
- Introduction - This section could be improved by including some specific examples of applications whenever possible. In particular, the concept seems a bit abstract since you do not really define "infection barcodes" or how you imagine infection history would be measured. For chronic viral infections (like HPV), maybe this is by sampling and genotyping at a single timepoint ? (though that might miss infections that have been cleared). For acute infections that induce strong strain-specific antibody responses, maybe it is assessed by specialized serological assays? More concrete examples of questions we might answer with this approach would also be helpful. For example, during the last big Zika outbreaks there was a lot of discussion about how Zika and dengue infections interacted immunologically... this method could clearly help with that questions.
  Thank you for this comment and for the detailed suggestions! We added a paragraph to better explain to a large audience how (and why) this inference method could be useful. In addition to the examples listed above, we also mention serology-driven studies (e.g. in the case of influenza).
- Methods

104-108 : A description of the model itself is missing here. This section describes the algorithm before the model. It would be helpful to first describe the model (ie SI/SIR/SIS/SIRS/SEIR, how multiple strains are handled, etc) and the possible transitions, in one section, before telling us how you implement it computationally, in a second section. For the first section, a model diagram and diagrammatic outline of the main paper idea would be very helpful.
We apologize for the lack of clarity. This is an event-based model implemented in a non-Markovian way so comparison with a classical ODE model can be misleading. However, we completely agree that there was room to clarify the life-history of the infection. This was done in the paragraph where we explain the Gillespie SSA and the various rates.
Page 4: It's a little confusing to use the letter "I" for these matrices since "I" usually means "infected"
We replaced the immunity matrix letter I by J.
Lines 129 - 136 : It would perhaps be helpful to give the variance of this distribution in terms of the parameters (as shape and scale are not particularly intuitive)
We added calculation for the variance for the default setting, which reads now: *... such that the mean was equal to one and the variance equal to* 2.27*, with* Γ *being the well-known gamma function.*
A population size of N=250 nodes seems very small and unrealistic for most infection scenarios of interest. Is the simulation method too computationally intensive to do anything larger? Do you anticipate that population size impacts the results at all?
The limitation in network size was indeed largely motivated by computational constraints. Indeed, since the model is non-Markovian, we need to keep track of the timing of all the events. Furthermore, the multiplicity of infections further increases the dimensionality of the problem. Also, high-quality data sets [Horby et al. 2021, Galanti et al. 2019, Murall et al.2019] that could potentially be used for validation including repeated measurements of multiple infections and detailed information on network properties consist typically of several hundred individuals only.

- Results
  It could be easier to interpret results if instead of just the highly clustered vs dispersed + non-clustered graphs, you also compared to one that was a close representation of a "well-mixed" population - e.g. a random uniform network
  We thank the reviewer for this suggestion. We had initially used Erdos-Renyi graphs as a comparator (data not shown). For this revision, we performed additional simulations on random regular graphs, where the degree is constant, and thus degree dispersion is zero. We added a figure with infection diversity and multiplicity to the supplement. We added the following sentences to the results section:
  $\rightarrow$ *In order to uncover potential biases stemming from degree dispersion and clustering, we also simulated multiple infections on random regular graphs, which had*

fixed degree of four and clustering coefficient of 0.014*.* ...

→ *Infection diversity on random regular networks mirrored in general those on clustered networks, with a markedly lower levels for partial cross immunity settings (Fig. S2).*

It would be helpful to consider providing more evidence to readers that the two networks compared in Table 1 are really a fair comparison. Generally for the same average degree, degree dispersal acts to increase R0 while clustering acts to decrease it, so it would seem that these networks would result in very different R0 values, rates of epidemic spread, and final epidemic sizes, even with single genotypes. And here average degree is not even constant. Have you compared spread and these summary statistics for an epidemic of a single genotype? It seems like you would want to be sure that you can isolate differences due to network type. The top and bottom row of Figure 1 do look pretty similar, but it's hard to tell by eye, and this is already including multiple strains.

We thank the reviewer for this excellent point. We performed the suggested simulations with only one genotype. As expected for a single genotype simulation, the immunity assumptions were obsolete. More interestingly, dispersed networks have larger median epidemic size (85%) **than clustered networks** (78%)**. Clustered networks have a large interquartile range, while dispersed networks show little variation around the median. For mean final epidemic size, this contrast is even stronger:** 86% **for dispersed and** 57% **for clustered networks. We added a supplementary figure to support the fact that the chosen networks would result in contrasting summary statistics for single infections. As expected, with multiple infections, diversity and multiplicity are constrained by immunological interference assumptions, and less so by the underlying network assumptions. We added the following paragraph:**

→ *To show that these networks would result in contrasting epidemic dynamics, we also considered single genotype simulations resulting in larger final epidemic size for dispersed networks (S1 Fig.).*

Most figure captions could be significantly expanded to clarify what is being shown in the figure, the definitions of the various quantities, and to remind the reader briefly of the methods

We apologize for this lack of details (which was also pointed by one of the reviewers) and have enriched the figure captions.

Figure 3: It might be helpful to report the R^2 values here, since that tells the reader how much of the variance in network path length between individuals can be explained by differences in their infection history

We thank the reviewer for this suggestion. We performed an anova on the model and calculated the variance explained by the barcode connectivity. We updated the figure with the results for each panel. Line 350: This is a bit unclear ... if the model does not include infection barcodes, then what does it include? **We apologize for being unclear here. The comparison is of course made w.r.t. random AUC of** 0.5 **as mentioned in the figure legend. We removed the part of the sentence referring to 'without using multiple infection data'**

- Discussion
  The authors could consider expanding on the assumptions behind their approach and what sort of data or types of infections would be necessary for this approach to be useful in the real world. It was a bit confusing when in Line 441 you mentioned this method was specific to PCR-based infection data, as that was not really clear from the rest of the paper.
  This is in line with one of the reviewers' concerns, which was about adding more details in the Introduction about how this method could be used practically.
  Regarding more specifically line 441 we clarified what we meant which is that the exact same method can be used to analyse serological data.
- Supplement
  Figure S2 is clearly for "dispersed networks", but is Figure S1 for "clustered networks"? Also these look like the same figure might be repeated accidentally? Figure S3 and S4 are missing Figure S5 - is this correct? Two figures appeared at the end without their captions
  The supplementary figures were renamed due to an additional figure. Figure S3 is indeed for clustered networks, and we added the description to the caption. Figures S3 and S4 are slightly different, they were not repeated accidentally. We also corrected the layout for the figures and captions for S5, S6, and S7.
